# Experimental validation of in silico analysis estimated the reverse effect of upregulated hsa‐miR‐106a‐5p and hsa‐miR‐223‐3p on *SLC4A4* gene expression in Iranian patients with colorectal adenocarcinoma by RT‐qPCR

**DOI:** 10.1002/cam4.5499

**Published:** 2022-12-05

**Authors:** Javad Ranjbaran, Hossein Safarpour, Samira Nomiri, Tahmine Tavakoli, Zohreh Rezaei, Fatemeh Salmani, Pegah Larki, Elham Chamani

**Affiliations:** ^1^ Department of Clinical Biochemistry, School of Medicine Birjand University of Medical Sciences Birjand Iran; ^2^ Cellular and Molecular Research Center Birjand University of Medical Sciences Birjand Iran; ^3^ Department of Internal Medicine, School of Medicine Birjand University of Medical Sciences Birjand Iran; ^4^ Department of Biology, Faculty of Sciences University of Sistan and Balouchestan Zahedan Iran; ^5^ Department of Epidemiology and Biostatistics, Social Determinants of Health Research Center, Faculty of Health Birjand University of Medical Sciences Birjand Iran; ^6^ Department of Molecular Genetics, Genomic Research Center Shahid Beheshti University of Medical Sciences Tehran Iran

**Keywords:** biomarker, colorectal cancer, hsa‐miR‐106a‐5p, hsa‐miR‐223‐3p, single‐cell, *SLC4A4*, WGCNA

## Abstract

**Background and Methods:**

Colorectal cancer (CRC) is considered one of the most common malignancies worldwide. The diagnosis and prognosis of the patients are very poor. In this study, we used in‐silico analysis and experimental techniques to investigate novel co‐expression genes and their associated miRNA networks in CRC. For this purpose, we conducted a comprehensive transcriptome analysis using online bulk and single‐cell RNA‐seq datasets. We then validated the results on tissue samples from cancerous and adjacent normal tissues from CRC patients by RT‐qPCR.

**Results:**

Using a weighted gene co‐expression network algorithm, we identified *SLC4A4* as a significantly downregulated hub gene in the CRC. The single‐cell analysis indicated that the expression level of *SLC4A4* in Paneth cells is higher than in other cell populations. Further computational analysis suggested hsa‐miR‐223‐3p and hsa‐miR‐106a‐5p as two specific hub‐miRNAs for the *SLC4A4* gene. RT‐qPCR analysis showed a 2.60‐fold downregulation of *SLC4A4*. Moreover, hsa‐miR‐223‐3p and hsa‐miR‐106a‐5p showed an increased expression level of 5.58‐fold and 9.66‐fold in CRC samples, respectively. Based on the marginal model analysis, by increasing the expression of hsa‐miR‐106a‐5p, the average expression of the *SLC4A4* gene significantly decreased by 103 units. Furthermore, ROC curves analysis indicated statistically significant for diagnostic ability of *SLC4A4* (AUC: 0.94, Sensitivity: 95.5%, Specificity: 95.5%) and hsa‐miR‐106a‐5p (AUC: 0.72, Sensitivity: 72.7%, Specificity: 100%).

**Conclusion:**

This study provides a framework of co‐expression gene modules and miRNAs of CRC, which identifies some important biomarkers for CRC pathogenicity and diagnosis. Further experimental evidence will be required to support this study and validate the precise molecular pathways.

## Graphical abstract

In this study, we identified biomarkers in colorectal cancer (CRC) in a comprehensive process by using the systems‐biology methods. At first, coding‐RNA and non‐coding‐RNA datasets were analyzed in the R software platform. Then the gene targets were identified with further analysis and appropriate filtering; finally, these gene targets were validated and confirmed in the tissues of CRC patients using the RT‐qPCR technique.

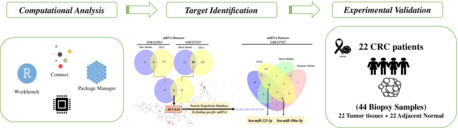

## 1 INTRODUCTION

Colorectal cancer (CRC) is a diverse disease defined by the progressive accumulation of genetic and epigenetic alterations.^1^ CRC is the third most harmful cancer and the fourth‐leading cause of cancer death worldwide.^2^ Despite this, research shows that CRC prognosis heavily depends on early detection and that cancer mortality can be reduced through proper monitoring and screening.^3^, ^4^ Early CRC diagnosis with high accuracy is important.

Screening permits diagnosis at an early stage of CRC, as well as a reduction in cancer mortality.^5^ Today, several tumor markers such as CEA, CA 242, and CA 19‐9 are used to diagnose and screen for CRC. Correspondingly, microRNAs (miRNAs), plasma‐based DNA, and stool proteins have been used as novel noninvasive screening in colorectal neoplasia. MiRNA expression abnormalities have been linked to various diseases, including cancer, and these tiny molecules are considered to play a role in cancer etiology.^6^, ^7^, ^8^

MiRNAs are a group of small non‐coding RNAs with about 19 to 25 nucleotides that inhibit mRNA translation and control gene expression.^9^, ^10^ Each miRNA can interact with various mRNAs, some of which may be repressed by other miRNAs.^11^

Hsa‐miR‐223‐3p and hsa‐miR‐106a‐5p as two potential specific miRNAs that can target the *SLC4A4* gene are known as carcinogenic miRNAs. *SLC4A4* (Solute Carrier Family 4 Member 4) is a membrane protein‐coding gene for a Na^+^/HCO3^−^ cotransporter.^12^ Expression of *SLC4A4* is likely to inhibit tumorigenesis via an ubiquitous mechanism. Its expression was found to have only minor correlations with *SMAD4* and *MUC4* mutations.^13^, ^14^ Inflammation‐driven colon cancer arises when *SMAD4* expression is lost in adenomas.^15^, ^16^

Using systems biology methods can lead to the discovery of key genes and miRNAs in an effective way to validate studies. Weighted Gene Co‐Expression Network Analysis (WGCNA) is a biology‐based, systematic, evolutionary method that explores fundamental transcriptome organization and identifies modules of highly correlated genes as biomarkers or therapeutic targets.^17^

Despite various research, the role of miRNA and genes in CRC remains unclear. As a result, the aim of this research is to discover novel molecular indicators in CRC as well as genetic targets for cancer diagnosis. First, we analyzed CRC‐related datasets using the WGCNA package in R software, and then used online databases to determine the mRNA‐miRNA relationship. Then, we confirmed the results in 44 tissue samples of Iranian patients with CRC in the laboratory to introduce the obtained genes and miRNAs as new biomarkers associated with CRC.

## 2 METHODS AND MATERIALS

**FIGURE 1:**
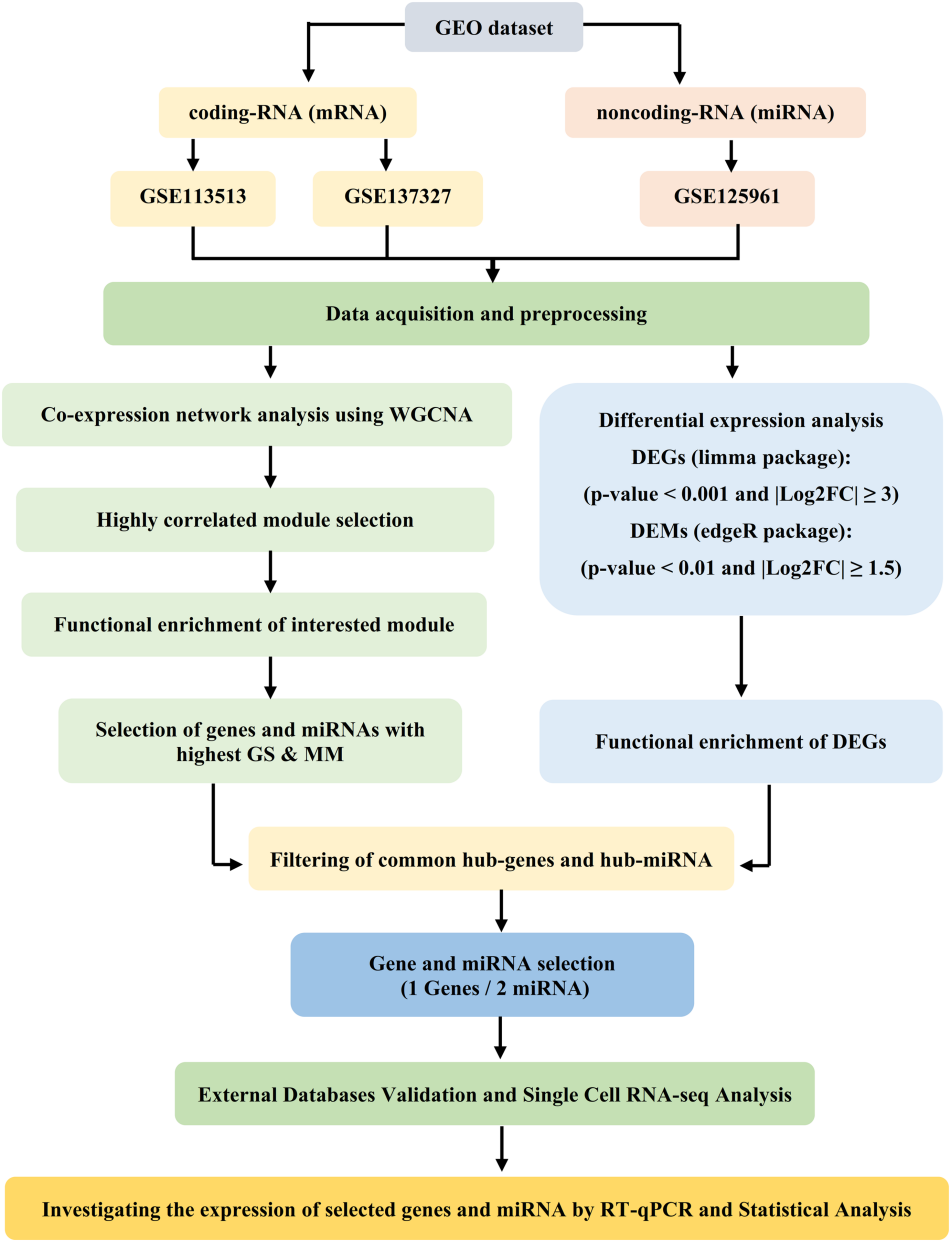
Overall process of the present study. Online expression data were downloaded from the GEO database and analyzed in two separate manners. To construct the co‐expression network, the WGCNA package was used and important modules were extracted. In parallel, DEG and DEM analyses were conducted to find the most variable genes and miRNAs through CRC development. Hub genes and hub miRNAs were selected based on being DEG (DEM) as well as a high degree of GS and MM values. Single‐cell analysis of CRC online data was used to characterize the expression behavior of interesting genes in different cell clusters. Finally, in silico results were validated on tissue samples from CRC and healthy individuals.

The schema of this study is shown in (Figure 1).

### 2.1 Systems biology approach

#### 2.1.1 Source and preprocessing of data

Data for RNA expression profiling of human CRC samples and adjacent normal tissues were received from the Gene Expression Omnibus (https://www.ncbi.nlm.nih.gov/geo/) database (Table S1). We filtered out the GEO database to find appropriate datasets based on the novelty of datasets, sample count, interesting tissue, sample types, and data quality. Raw data were normalized and adjusted, and probe IDs were converted to gene symbols. The top 4000 genes based on the coefficient of variation (CV) values were then selected for subsequent analyses.

#### 2.1.2 Differentially expressed genes and miRNAs

The Limma package on GEO2R online software and the edgeR package were used to investigate DEGs (Differentially Expressed Genes) and DEMs (Differentially Expressed miRNAs) between CRC and adjacent normal tissues. The following criteria were applied to filter the genes with the peak differentially expressed: for DEGs: *p‐*value <0.001 and |Log2FC| ≥ 3; and for DEMs: *p‐*value <0.01 and |Log2FC| ≥ 1.5. The DEGs were then examined for Gene Ontology (GO) and Kyoto Encyclopedia of Genes and Genomes (KEGG) pathway enrichment using the Enrichr database (https://maayanlab.cloud/Enrichr/). *p*‐value<0.05 indicated significant enrichment.

#### 2.1.3 Network construction and key module identification

We used WGCNA to rebuild the co‐expression network of CRC RNAs. Using a Pearson correlation matrix, a co‐expression analysis for paired genes was organized. Following that, the appropriate *β‐*value was considered based on a topological algorithm for the adjacency matrix that fulfill the criteria of scale‐free co‐expression network topology.^18^, ^19^ Afterward, TOM similarity and dissimilarity modules are used to establish a Topological Overlap Matrix (TOM) and dissimilarity TOM (dissTOM) for the next step. Finally, the module identification was done using a dynamic tree cut with a specified size of at least 30 modules. For each dataset, a threshold value was used to merge modules with high similarity scores.

#### 2.1.4 Functional enrichment analysis and construction of MTR (module‐trait relationships)

The aim was to find modules that were strongly associated with the clinical features that were assessed. For this purpose, using Module Eigengene (ME), the expression profiles of each module were briefed as the eigenvector related to the first principal component of the expression matrix. Moreover, the gene significance (GS) index was used to estimate the association of individual RNAs with the CRC. Module Membership (MM) was also defined as the ME correlation and each module's RNA expression profile. The most essential (central) parts in the modules are likewise tightly related to the characteristics of GS and MM are substantially linked.^20^ They may be utilized to build a network and find hub‐genes and hub‐miRNAs.

The Enrichr database was used to perform gene enrichment analysis in the selected module. “GO Biological Process 2018” and “KEGG 2019 human” databases were specified for pathway enrichment and functional analyses. A Bonferroni significance limit of 0.05/nMods, where nMods is the number of modules belonging to the given dataset, was used to assess the importance of GO and KEGG pathway keywords.

#### 2.1.5 Determination of co‐expression network reconstruction

Those genes or miRNAs with the highest GS (Gene Significance) and MM (Module Membership) were selected as hub genes or hub‐miRNAs when differently expressed from the control samples. Then, using the web tool “Venny” v 2.1, a Venn diagram was created (https://bioinfogp.cnb.csic.es/tools/venny/) to find out the similarity of gene lists. GeneMANIA (https://genemania.org) created co‐expression networks, which are hub genes from different modules and their co‐expressed neighbor genes.

In parallel, a list of specific miRNAs for selected hub‐gene was downloaded from TargetScan databases (http://www.targetscan.org/vert_72/ version 7.2 (March 2018)) as a resource for experimentally validated miRNA‐target interactions.

#### 2.1.6 External database validation of selected hub‐gene

The GEPIA online database (http://gepia.cancer‐pku.cn) was utilized to investigate the influence of the chosen hub gene on overall survival (OS) in CRC patients and compare the expression level of filtered genes in healthy and CRC groups. UCSC Cancer Browser (https://xenabrowser.net/ (accessed on 18 May 2021)) was used to collect data from the TCGA‐COAD database for the first confirmation of chosen hub‐gene expression in primary tumors, normal solid tissue, recurrent tumors, and metastatic cancers. Then, using the Xena browser, the expression of a particular hub‐gene in primary and metastatic cells was acquired from the Cancer Cell Line Encyclopedia (CCLE) databases.

#### 2.1.7 Single‐cell RNA‐seq analysis of CRC samples

Then, in all subtypes of colorectal tissues, we completed single‐cell transcriptome analyses to define cell‐type‐specific molecular markers of CRC. We obtained the raw single‐cell RNA‐seq data from Gene Expression Omnibus (GEO) under the access code GSE163974 (Table S1). Researchers in the original study applied single‐cell transcriptomics to simultaneous measurement of telomere length and transcriptome in the same cells to enable systematic assessment of cancer stem cells (CSCs) in primary CRC. The Scanpy toolkit^21^ was leveraged for data analysis. First, low‐quality cells were filtered using the quality control step. Cells that satisfied the following criteria were kept in this process: More than 500 genes, fewer than 17,500 counts, and <20% of reads mapped to mitochondrial genes. The normalized total function in scanpy, or the calculateSumFactors function from SCRAN, predicts size factors for each cell to reduce bias within cell counts and boost cross‐cell comparison values of cell expression. It is used to compute normalized expression. To facilitate unsupervised clustering and cell‐type identification, dimensionality reduction was performed with the top 4000 highly variable genes for principal component analysis (PCA). Following the identification of highly variable genes, PCA was run on the combined set of data for each sample. We created a closest neighbor graph for each cell after we were embedded in this PCA space, identifying the *k* = 15 nearest neighbors for each cell. Using a minimum distance of 0.5 and a spread of 1.0, we created a uniform manifold approximation (UMAP) embedding for visualization from this closest neighbor graph.^22^ Afterward, Louvain community detection^23^ was used to define a cluster partition by the nearest neighbor graph constructed in PCA space. Cell types were recognized based on the expression levels of marker genes from the literature.

### 2.2 Experimental validation

#### 2.2.1 Patients and demographic

Twenty‐two CRC Patients referred to the Gastroenterology and Liver Diseases Research Center of Shahid Beheshti University of Medical Sciences between June 2017 and November 2018 provided the colon and rectal tissues (22 cancers and 22 adjacent normal tissues). Inclusion criteria for these patients were genetic and pathological confirmation. Patients having underlying illnesses, such as diabetes, autoimmune disease, cardiovascular disease, chemotherapy, or radiation history, were not included in the study. Furthermore, based on the Helsinki Declaration, which was accepted by the ethics council of Birjand University of Medical Sciences, ethical concerns and patient satisfaction were observed (IR.BUMS.REC.1399.371). Demographic information about patients was presented in (Table S5).

#### 2.2.2 RT‐qPCR evaluating

To validate the in silico results, reverse‐transcription‐quantitative‐real‐time PCR (RT‐qPCR) analyses were conducted for CRC tissues and adjacent normal. Total RNA was extracted from samples (average weight 45 mg) using TRIzol reagent (Invitrogen; Thermo Fisher Scientific, Inc., USA, Cat No: 15596026). Total RNA was reverse transcribed into cDNA using the RevertAid First Strand cDNA Synthesis Kit (Thermo Fisher Scientific, USA, Cat No: K1622) for gene expression assay and the miScript II RT kit (Qiagen, Germany, Cat No: 218161) for miRNA expression assay according to the manufacturer's instructions after quantification and qualification of RNA samples using the NanoDrop spectrophotometer (Epoch BioTek). After that, RT‐qPCR was performed using RealQ Plus Master Mix Green high ROX (Amplicon, Denmark, Cat No: 5000830) and an Applied Biosystems 7500 Real‐time PCR system (Thermo Fisher Scientific, Inc., Waltham, MA, USA). The primer sequences applied in RT‐qPCR are presented in (Table S6). In this step, *GAPDH* and U6 RNA acted as endogenous controls for estimating the expression levels to standardize the relative expression levels for data analysis. The data were then analyzed using the 2^−ΔΔCT^ method. Each sample was examined in triplicate.

#### 2.2.3 Statistical analysis

RT‐qPCR results were analyzed using SPSS, and plots were designed with GraphPad Prism Software (versions 16 and 8, respectively). The Shapiro–Wilk normality test was then performed to compare the normality of the variables between the two study groups. After that, an unpaired Student's *t*‐test was used to determine the statistical significance of disagreement among normally distributed data. A *p*‐value <0.05 was considered statistically significant. Moreover, coefficient estimation of the marginal model was conducted for the prediction of selected hub‐gene changes. Moreover, the Receiver Operating Characteristics (ROC) curve analysis was conducted to assess the sensitivity and specificity of hub‐gene and hub‐miRNA to determine the CRC diagnosis index.

## 3 RESULTS

### 3.1 Preprocessing data and detection of DEGs and DEMs

**FIGURE 2:**
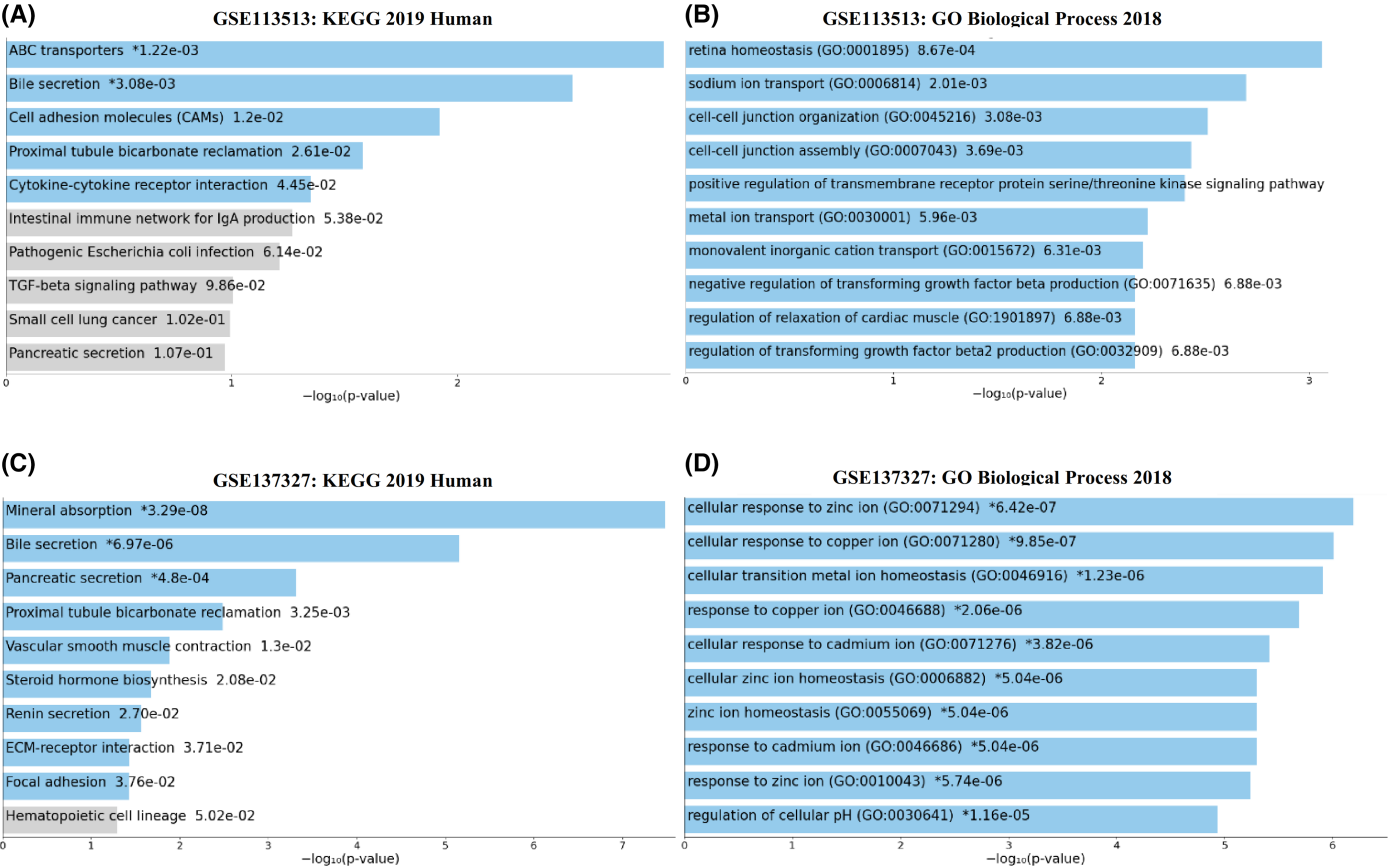
Functional enrichment analysis of DEGs using the Enrichr database. (A) and (B) GSE113513 KEGG and BP results; (C) and (D) GSE137327 KEGG and BP results.

Three expression datasets containing CRC and control samples were used to investigate the role of coding and non‐coding RNAs in CRC Table S1. To lessen the influence of technical noise in each expression data set, quantile normalization was used. Sample clustering did not show any outliers in the mRNA and miRNA datasets (Figure S1A–C), and both were used in subsequent analyses. In the next step, DEGs and DEM analyses were performed (Table S2). The enrichment results of two mRNA datasets using the Enrichr database (https://maayanlab.cloud/Enrichr/) are shown in (Figure 2). The most significant pathways associated with GSE113513 DEGs were ABC transporters, bile secretion, cell adhesion molecules, and cytokine‐cytokine receptor interaction. On the other hand, retina homeostasis, sodium ion transport, and cell–cell junction organization were the most important biological functions of GSE113513 DEGs (Figure 2A,B). Bile and pancreatic secretion, and mineral absorption were found to be the most significant biological pathways for DEGs in the GSE137327 dataset. The biological processes of DEGs for this dataset included cellular response to zinc ion, cellular transition metal ion homeostasis, and cellular response to copper ion (Figure 2C,D).

### 3.2 Identifying clinically important modules

**FIGURE 3:**
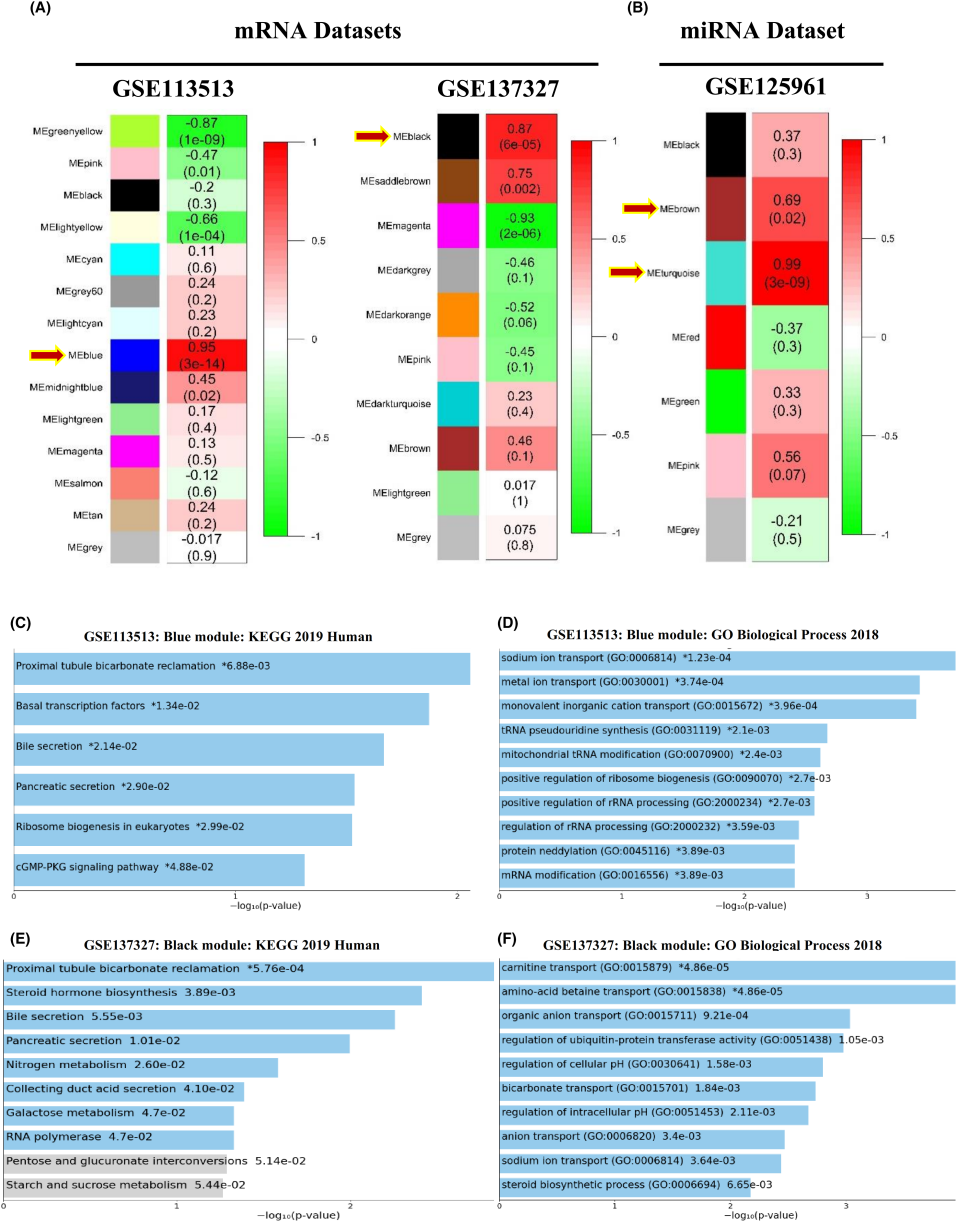
Module Trait Relationship and enrichment analysis of interesting modules. (A) The best MTR of mRNA datasets; (B) The best MTR of miRNA datasets; (C) and (D) KEGG and BP enrichment results from GSE113513 blue module; (E) and (F) KEGG and BP enrichment results from GSE137327 black module. MTR, Module Trait Relationship.

With a cutoff R^2^ value of 0.9, soft threshold *β* values of 10 and 7 were selected for GSE137327 and GSE113513, respectively, while a value of 10 was chosen for GSE125961 (Figure S2A–C). Because these values follow the power‐law distribution, the networks are closer to the true biological network state. The results of the hierarchical clustering analysis based on weighted correlation were then separated according to the stipulated criteria for creating gene modules. To integrate the modules, the minimum module size was set to 30 with a height cut of 0.25 (Figure S3A–C). In Figure 3, all gene co‐expression modules were visualized. We choose modules with the highest positive correlation for downstream analysis. These modules are listed in Table S3.

### 3.3 Hub mRNA and miRNA detection

**FIGURE 4:**
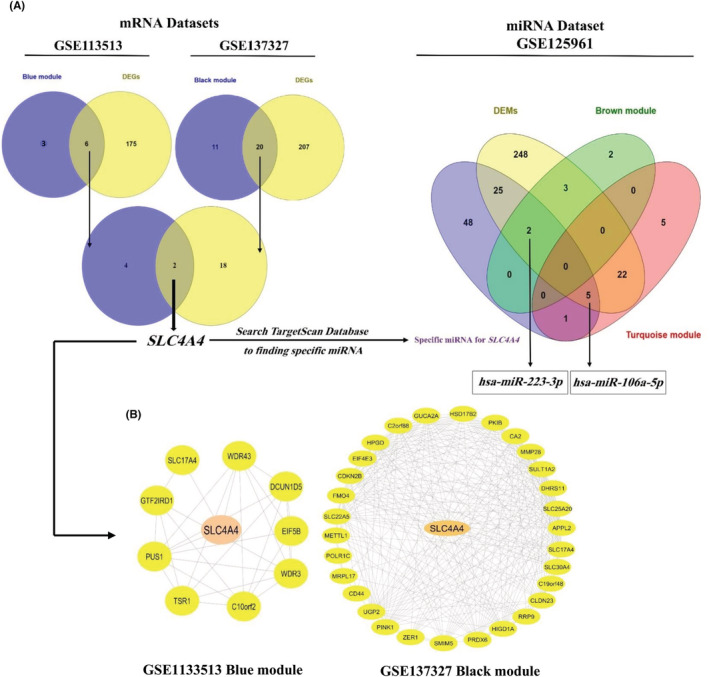
Selection of genes, miRNAs, and co‐expression networks. (A) hub‐mRNA and hub‐miRNAs selection process by systems biology approach; (B) The co‐expression networks of *SLC4A4* in Blue module of GSE1133513 and Black module of GSE137327. DEG: Differentially expressed gene; DEM: Differentially expressed miRNA.

The overall process of hub mRNAs and miRNA selection is visualized in (Figure 4A). First, mRNAs with the maximum MM (Module Membership) and GS (Gene Significance) values in each module (Figure S4A–D) were compared with their DEG list counterparts, and the overlapping genes were then considered the final hub genes. In miRNAs, those miRNAs with maximum MM and GS were compared with the DEMs list and screened based on a list of specific miRNAs from the TargetScan database. The filtered miRNAs were then considered the final hub miRNAs (Figure 4A). Regarding the applied filters, from the two genes which met the criteria, *SLC4A4*; and from 32 miRNAs, hsa‐miR‐223‐3p and hsa‐miR‐106a‐5p were selected for downstream analysis. Finally, the co‐expression network of the black module of GSE137327 and the blue module of GSE113513 was reconstructed using GeneMANIA and Cytoscape software (Figure 4B).

### 3.4 Characterization of *SLC4A4* expression by external databases

**FIGURE 5:**
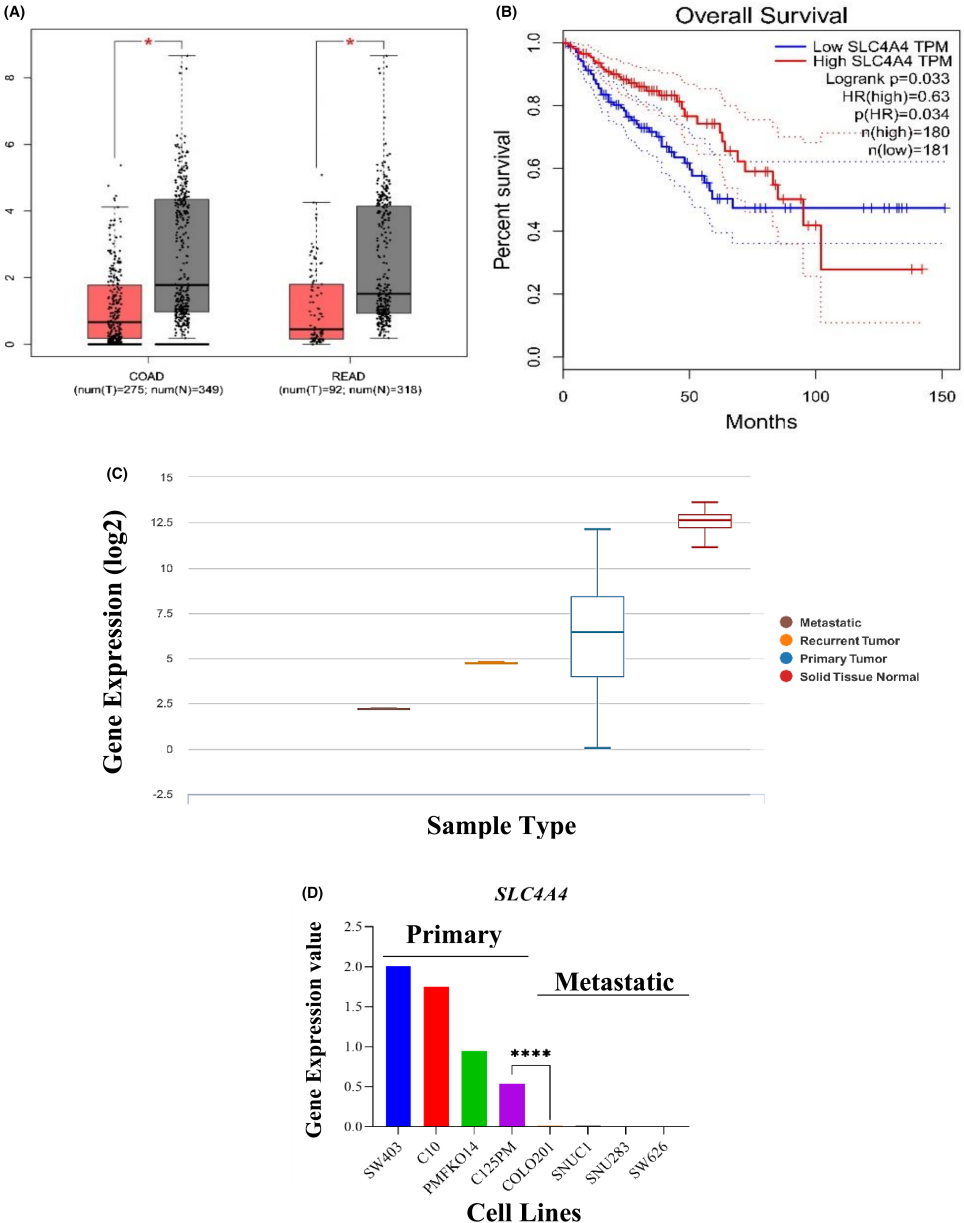
Characterization of *SLC4A4* gene expression using external databases. (A) Differential expression of the *SLC4A4* gene in colon (*n* = 624) and rectum (*n* = 410) samples using GEPIA database. *SLC4A4* expression was decreased in tumor samples compared to controls; (B) The overall survival rate of high‐ and low‐risk patients based on the expression levels of *SLC4A4* in the GEPIA database. As shown, downregulation of the *SLC4A4* gene has a significant impact on overall survival in CRC patients; (C) *SLC4A4* expression profiles and clinicopathological data of patients with CRC. *SLC4A4* gene expression was downregulated in the primary tumor, metastatic and recurrent tumor compared to solid tissue normal samples (*p* < 0.0001); (D) The CCLE showed a high level of *SLC4A4* expression in primary (SW403, C10, PMFKO14, and C125PM) compared to metastatic (COLO201, SNUC1, SNU283, and SW626) cell lines.

To verify *SLC4A4* gene expression, we used GEPIA, which contained TCGA and GTEx CRC samples. (Figure 5A) indicated the expression level of the *SLC4A4* gene in COAD and READ samples of CRC. The results showed a significantly decreased level of *SLC4A4* expression in tumor samples compared to controls. It was also revealed that downregulation of *the SLC4A4* gene has worse overall survival in CRC patients (Figure 5B). The UCSC Xena browser (https://xenabrowser.net/ (accessed on 18 May 2021)) was used to get clinicopathological data from individuals with primary CRC. As shown in (Figure 5C), *SLC4A4* expression was significantly different in solid tissue normal (N = 41), primary (*N* = 286), recurrent tumors (*N* = 1), and metastatic tumors (*N* = 1). Our results showed that *SLC4A4* gene expression was downregulated in the primary tumors, metastatic and recurrent tumor compared to solid tissue normal samples (*p* < 0.0001). Inconsistent with the above results, according to the CCLE database (https://portals.broadinstitute.org/ccle), *SLC4A4* expression was substantially higher in the primary (SW403, C10, PMFKO14, and C125PM) compared to metastatic (COLO201, SNUC1, SNU283, and SW626) cell lines (Figure 5D).

### 3.5 Single‐cell transcriptome analysis

**FIGURE 6:**
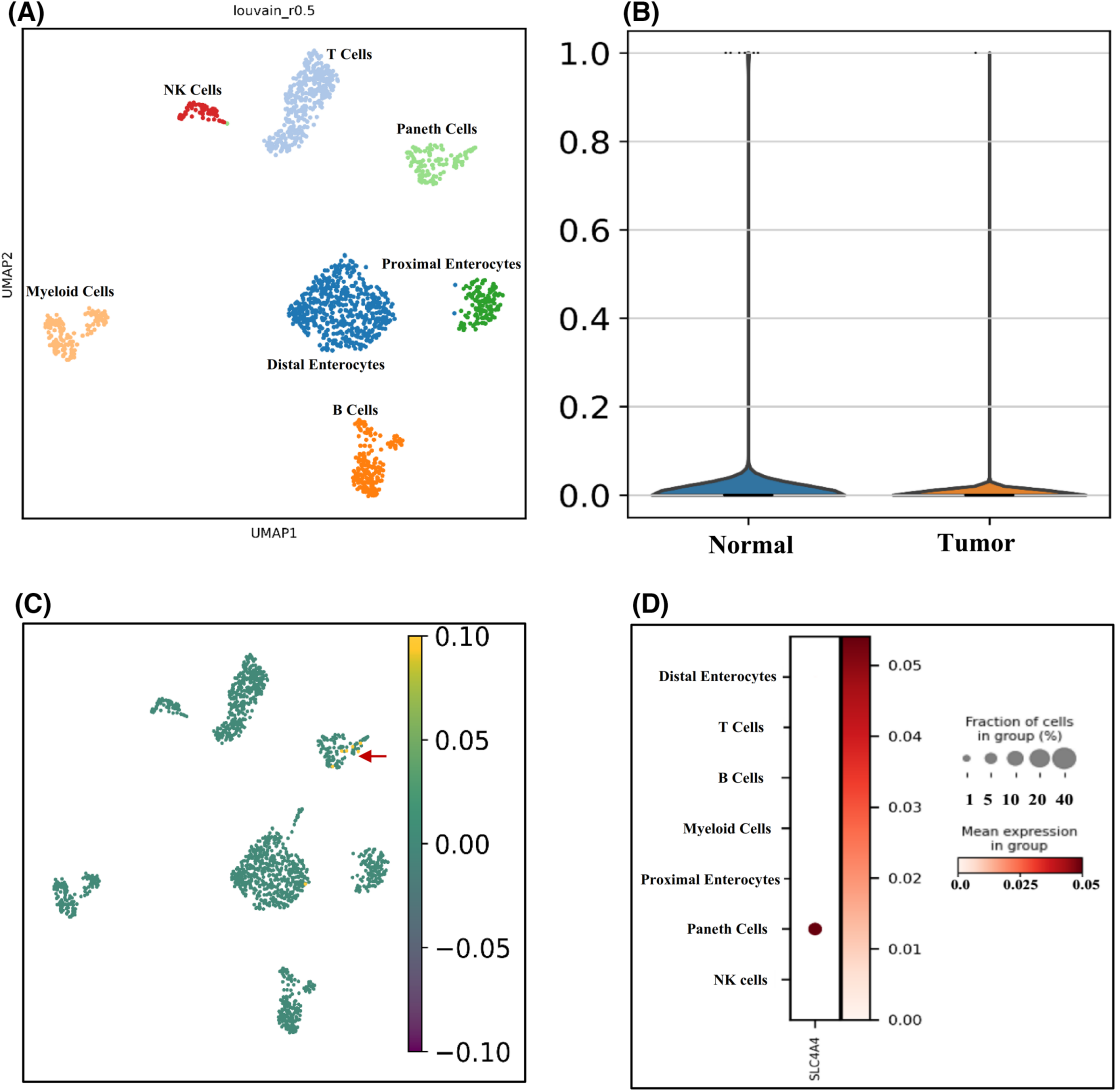
Transcriptomics comparison of CRC and control CSCs. The UMAP projection of the cell in the normal situation (*n* = 1456) was visualized in dark blue, and the cell in the CRC sample (*n* = 754) was visualized in light blue. (A) Louvain clustering and cell annotations were used to identify seven major cell populations; (B) *SLC4A4* equation with different status of CSC from CRC sample; (C) *SLC4A4* expression in various cell populations of CSC from CRC samples; (D) Dot plot of *SLC4A4* expression in different clusters.

Wang et al. recently reported the molecular signature of CRC pathogenicity in cancer stem cells (CSCs) using single‐cell RNA sequencing technologies. As most of the cells in this dataset are CSCs cells, this dataset offers a precious resource for understanding the expression situation of our target gene (*SLC4A4*), which was selected from previous sections. We used the latest development package Scanpy^21^ to reanalyze scRNA‐seq data. A total of 1527 single‐cell transcriptomes were accepted after stringent quality control measures. The Louvain clustering and cell annotation were employed to identify major cell populations. We evaluated the distribution of cell numbers for each cluster by comparing the total number of cells from the CRC group to controls. As shown in (Figure 6A), seven different types of cells were clustered based on the specific markers between control and CRC patients (Table S4).

### 3.6 Visualization of *SLC4A4* in a single‐cell resolution

To understand the gene expression behavior of *SLC4A4* in different cell types, the expression of this gene was visualized using UMAP. Figure 6B represents the expression level of the *SLC4A4* gene between the normal and tumor samples. Referring to this figure, the potential of Paneth cells for *SLC4A4* expression is higher than in other cell populations (Figure 6C,D).

### 3.7 Genes expression status by RT‐qPCR and ROC curve

**FIGURE 7:**
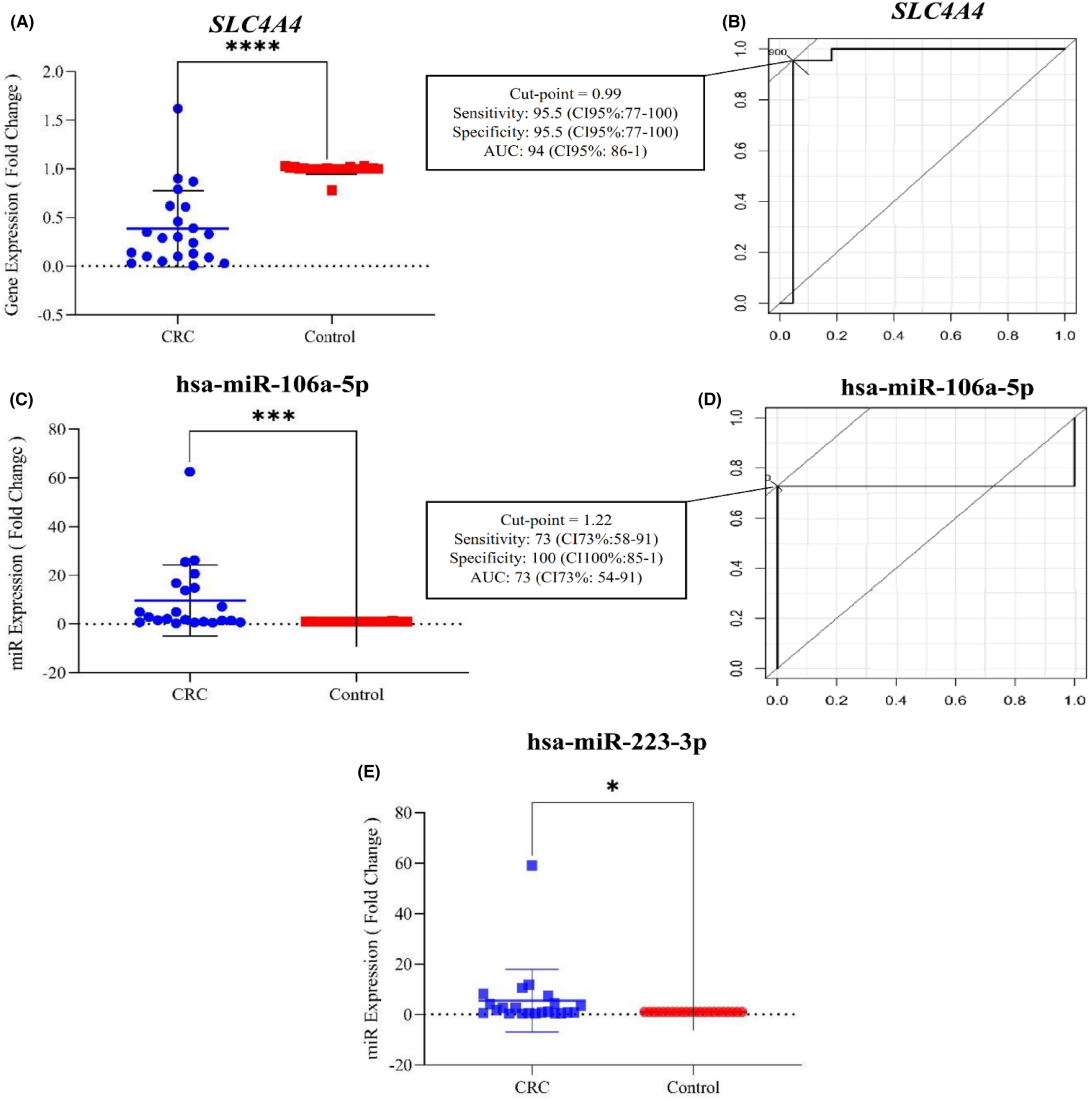
RT‐qPCR and ROC curves analysis of *SLC4A4*, hsa‐miR‐106a‐5p and hsa‐miR‐223‐3p expressions status. (A) *SLC4A4* is downregulated significantly in tumor tissues compared with normal tissues (*p*‐value = 0.0001); (B) *SLC4A4*; The ROC curves of the external validation indicated statistically significant for diagnostic ability of *SLC4A4* (AUC: 0.94, Sens: 95.5%, Spec: 95.5%, *p*‐value = 0.0001); (C) hsa‐miR‐106a‐5p showed significant elevated expression in tumor vs normal samples (*p*‐value = 0.0008); (D) hsa‐miR‐106a‐5p; The ROC curves of the external validation indicated statistically significant for diagnostic ability of hsa‐miR‐106a‐5p (AUC: 0.72, Sens: 72.7%, Spec: 100%, *p*‐value = 0.0098); (E) hsa‐miR‐223‐3p showed significant increased expression in tumor vs normal samples (*p*‐value = 0.046).

RT‐qPCR of *SLC4A4*, hsa‐miR‐106a‐5p, and hsa‐miR‐223‐3p was performed using cDNA from CRC and control tissue samples to validate in silico analysis results. According to Figure 7A, the expression of *SLC4A4* in the CRC sample was significantly reduced compared to the control (2.60‐fold down, *p*‐value = 0.0001). The ROC curve for *SLC4A4* showed significant diagnostic potential (AUC: 0.94, *p*‐value <0.001) with 95.5% sensitivity and specificity. A cutoff point of 0.9 for the *SLC4A4* gene indicates that this gene can distinguish patient tissues from healthy tissues (Figure 7B). Furthermore, the increased expression of hsa‐miR‐106a‐5p (9.66‐fold up, *p*‐value = 0.0008) is shown in (Figure 7C). Based on ROC curve analysis, hsa‐miR‐106a‐5p can also be considered as a diagnostic marker (AUC: 0.72, *p*‐value = 0.01) with a sensitivity and specificity of 72.7 and 100%, respectively. With a cutoff point of 1.22, 72% of cases can be correctly distinguished (Figure 7D). However, further research with larger sample sizes is needed to prove its effectiveness. At last, we found that hsa‐miR‐223‐3p was significantly upregulated in CRC samples (5.58‐fold up, *p*‐value = 0.0462) (Figure 7E). But ROC curve analysis failed to show any potential diagnostic value for hsa‐miR‐223‐3p (AUC: 0.54, *p*‐value = 0.64) (data not shown).

### 3.8 Statistical and marginal model analysis

The average age of the samples was 61.45 ± 11.48 years. 58% were male and 30% had a family history of cancer. All patients had adenocarcinoma. The highest incidence of malignancy was in the rectal region 40% and in the transverse colon 20% (Table S5). Due to the non‐normality of expression results, Box‐Cox conversion was used to create normality, and the marginal model results were accordingly fitted. The results of marginal model analysis with the Generalized Estimating Equations (GEE) approach indicated that the expression level of *SLC4A4* in the tumor tissues is reduced compared to the healthy tissues (Model 1: *b* = −0.28, *p*‐value <0.001 and Model 2: *b* = −0.34, *p*‐value <0.001). Furthermore, by increasing hsa‐miR‐106a‐5p, the average expression of *SLC4A4* gene decreased significantly by 103 units (Model 1: *b* = −0.103, *p*‐value = 0.01) (Table 1).

**TABLE 1:** Coefficient's estimation of marginal model for prediction of *SLC4A4* change

	Parameter	Coefficients	SE	Test statistics	*p*‐value
Model 1	CRC (base line = control)	−0.281	0.0671	17.540	0.000
	hsa‐miR‐106a‐5p.3^(1/3)^	−0.103	0.0414	6.131	0.013
Model 2	CRC (base line = control)	−0.341	0.0626	29.760	0.000
	hsa‐miR‐223‐3p.3^(1/3)^	−0.034	0.0592	0.329	0.566

## 4 DISCUSSION

Traditional single‐gene research has significant limitations in elucidating the complicated signaling network of tumor genes. WGCNA is now being used for data mining in various malignant tumors. By combining clinical information analysis, a series of modules associated with tumor grading, staging, and prognosis is achieved. Furthermore, key genes inside the modules are mined further to discover genes that affect tumor incidence, development, and metastasis.^17^ We focused on co‐expression network analysis to infer biological functions and diagnostic biomarkers for CRC.

Our in silico analysis identified *SLC4A4* as a significantly downregulated hub‐gene and associated biomarker in Iranian CRC tissues. Moreover, our systems biology and RT‐qPCR results supposed the *SLC4A4* gene is a potential target for upregulated hsa‐miR106a‐5p and hsa‐miR‐223‐3p in CRC samples. It is possible that these two miRNAs, by trapping *SLC4A4* mRNA or destroying it through near‐perfect complementary and partial complementary binding mechanisms,^24^ prevented the *SLC4A4* expression and its protein translation stage, which resulted in the reduction of the cytoplasmic level of this gene.

*SLC4A4* gene as a Na^+^/HCO3^−^ cotransporter, mainly involved in the secretion and absorption of sodium bicarbonate. This process is essential to maintain the dynamic pH equilibrium within cells.^12^ Furthermore, studies showed that *SLC4A4A* expression was associated with steroid biosynthesis, mismatch repair, base excision repair, DNA replication, and proteasome biological pathways.^25^ Thus, *SLC4A4* is involved in the suppression of tumorigenesis and the development of cancer and has important diagnostic and therapeutic implications.^16^

In silico analysis suggests that downregulation of *SLC4A4* expression can affect several biological processes, including mismatch repair, base excision repair, DNA replication, poor prognosis, invasion, and metastasis.^25^ Moreover, univariate and multivariate analysis revealed that *SLC4A4* was related to the overall survival (OS) of CRC patients.^26^, ^27^, ^28^, ^29^ On the other hand, upregulation of *SLC4A4* can decrease cell proliferation, migration, invasion, and inhibit apoptosis of CRC cells.^30^

However, in a recent study by Zelin Liu et al. (2022), *SLC4A4* knockdown in prostate cancer (PCa) inhibited cell proliferation, migration, and invasion while facilitating apoptosis. Also, overexpression of *SLC4A4* in tumor specimens correlated with disease progression via the AKT‐mediated signaling pathway.^31^ These findings might be explained by the characteristics of the tissue under investigation and the role of this gene in the tissue's particular signaling pathways.

Xiao et al. (2019) used a luciferase assay to confirm that hsa‐miR‐223 directly targeted the *SLC4A4* gene and indicated the downregulation of the *SLC4A4* expression in kidney cancer.^32^ Consistent with their results, we also confirmed the downregulation of *SLC4A4* gene and upregulation of hsa‐miR‐223‐3p in Iranian CRC patients by employing in silico and RT‐qPCR analysis. Moreover, recent studies indicated that hsa‐miR‐223‐3p as an onco‐miRNA could suppress *SLC4A4/KRAS* signaling pathways and lead to cancer malignancy in kidney and pancreatic cancers.^32^, ^33^ Thus, some bacterial species like *Helicobacter pylori* can lead to the induction of hsa‐miR‐223‐3p expression in patients with gastric cancer.^34^ On the other hand, hsa‐miR‐223‐3p can be considered a good prognostic marker in cancer diagnosis.^35^, ^36^

Our marginal model analysis (based on RT‐qPCR results) indicated that upregulation of hsa‐miR‐106a‐5p reduced the average expression of the *SLC4A4* gene by 103 units. This finding is valuable because, this miRNA is a member of the most common group of onco‐miRNAs, which, were suggested as an important candidate for diagnostic biomarkers in cancers.^37^ The genes which are under the control of this miRNA are involved in the cell cycle and PI3K‐Akt, FoxO, and p53 signaling pathways.^38^, ^39^ Peng Q et al. (2020) revealed that measuring miR‐106a is a promising biomarker for diagnosing and predicting survival in CRC patients.^40^ In this in silico‐based study, the patients with higher expression levels of miR‐106 had significantly shorter survival. Gene Ontology and signaling pathway analysis indicate that the miR‐106 family plays a significant role in the onset and progression of CRC. Therefore, recent publications suggest the prognostic role of hsa‐miR‐106a in CRC progression.^40^, ^41^ Furthermore, we could propose that the hsa‐miR‐106a/*SLC4A4* regulatory network plays a role in CRC pathogenicity.

The current study has some limitations which need to be noted. The first is the small sample size utilized in the experimental validation section; the results would be more reliable if more patients were available. Second, further experimental research should be carried out to investigate the functional role of the genes discovered in this work and confirm their expression at the serum and protein levels.

## 5 CONCLUSION

Based on this study, we confirmed the downregulation of *SLC4A4* expression and upregulation of hsa‐miR‐223‐3p and hsa‐miR‐106a‐5p, which may be regarded as a regulatory network in CRC tumor samples. Thus, based on the results of the ROC curve and statistical analysis, *SLC4A4* and hsa‐miR‐106a‐5p are suggested as diagnostic markers in CRC tissues. However, further experimental evidence will be required to support this study and validate the precise molecular pathways.

## AUTHOR CONTRIBUTIONS

**Javad Ranjbaran:** Conceptualization (supporting); data curation (supporting); investigation (lead); methodology (supporting); software (lead); validation (lead); visualization (equal); writing – original draft (equal); writing – review and editing (equal). **Hossein Safarpour:** Conceptualization (lead); data curation (lead); formal analysis (equal); investigation (equal); software (equal); supervision (equal); validation (equal); writing – original draft (equal); writing – review and editing (equal). **Samira Nomirii:** Formal analysis (equal); investigation (equal); validation (equal); visualization (equal); writing – original draft (equal); writing – review and editing (equal). **Tahmine Tavakoli:** Funding acquisition (supporting); validation (supporting); visualization (supporting). **Zohreh Rezaei:** Conceptualization (supporting); investigation (supporting); validation (supporting); writing – original draft (supporting). **Fatemeh Salmani:** Methodology (supporting); software (supporting); validation (supporting). **Pegah Larki:** Investigation (supporting). **Elham Chamani:** Conceptualization (lead); data curation (lead); funding acquisition (lead); validation (supporting); visualization (supporting); writing – original draft (supporting); writing – review and editing (supporting).

## FUNDING INFORMATION

No funding.

## CONFLICT OF INTEREST

The authors declare that they have no known competing financial interests or personal relationships that could have appeared to influence the work reported in this paper.

## ETHICS APPROVAL AND CONSENT TO PARTICIPATE

All tissues were obtained from the Gastroenterology and Liver Diseases Research Center biobank of Shahid Beheshti University of Medical Sciences, and Informed consent was obtained from patients before the sampling procedure. Also, Ethical considerations and patients' satisfaction were observed based on the Helsinki Declaration, which was approved by the ethics committee of Birjand University of Medical Sciences (IR.BUMS.REC.1399.371). Ethics certification is available on the Iran National Committee for Ethics in Biomedical Research website under the link: https://ethics.research.ac.ir/ProposalCertificateEn.php?id=162268&Print=true&NoPrintHeader=true&NoPrintFooter=true&NoPrintPageBorder=true&LetterPrint=true

## Supporting information


Figure S1–S4

Table S1–S6

## Data Availability

N/a
