# Supplementary material for: Experimental validation of in silico analysis estimated the reverse effect of upregulated hsa‐miR‐106a‐5p and hsa‐miR‐223‐3p on SLC4A4 gene expression in Iranian patients with colorectal adenocarcinoma by RT‐qPCR
Source: Cancer Med. 2022 Dec 5;12(6):7005–18. doi: 10.1002/cam4.5499 (PMC10067115; doi:10.1002/cam4.5499)
Supplement: Supplementary file 1 — Figure S1–S4 [file CAM4-12-7005-s001.docx]

**Supplementary figure legends:**

**Figure S1.** Sample clustering to detect outliers. Sample dendrogram and trait heatmap of **(A)** GSE113513; **(B)** GSE137327; **(C)** GSE125961.

**Figure S2.** Selection of the soft-thresholding powers. The left panel shows the scale-free fit index (y-axis) as a function of the soft-thresholding power (x-axis). The right panel displays the mean connectivity (degree, y-axis) as a function of the soft-thresholding power (x-axis). **(A)** GSE137327; **(B)** GSE113513 and **(C)** GSE125961.

**Figure S3.** Cluster dendrogram and module assignment from WGCNA. The branches correspond to highly interconnected groups of genes. Colors in the horizontal bar represent the modules. **(A)** GSE137327; **(B)** GSE113513 and **(C)** GSE125961.

**Figure S4.** Module features of GS and MM (a) Modules significantly correlated with AA status (control vs. patient). Each point represents an individual gene within each module, which are plotted by GS on the y-axis and MM on the x-axis. **(A)** GSE137327 (blue module); **(B)** GSE113513 (black module); **(C)** GSE125961 (turquoise module) and **(D)** GSE125961 (brown module).

**FIGURE S1**

**Figure S3.** Sample clustering to detect outliers. Sample dendrogram and trait heatmap of **(A)** GSE113513; **(B)** GSE137327; **(C)** GSE125961.


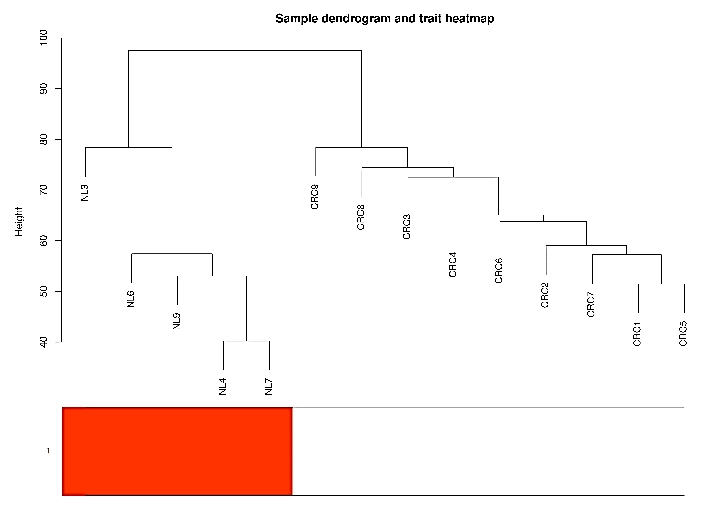

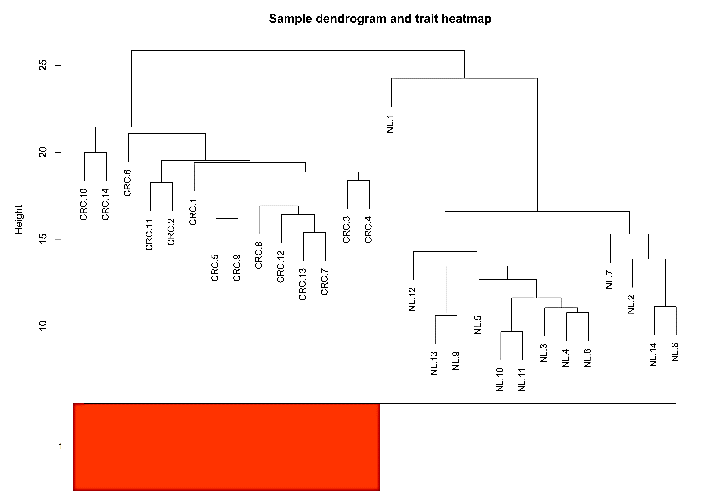

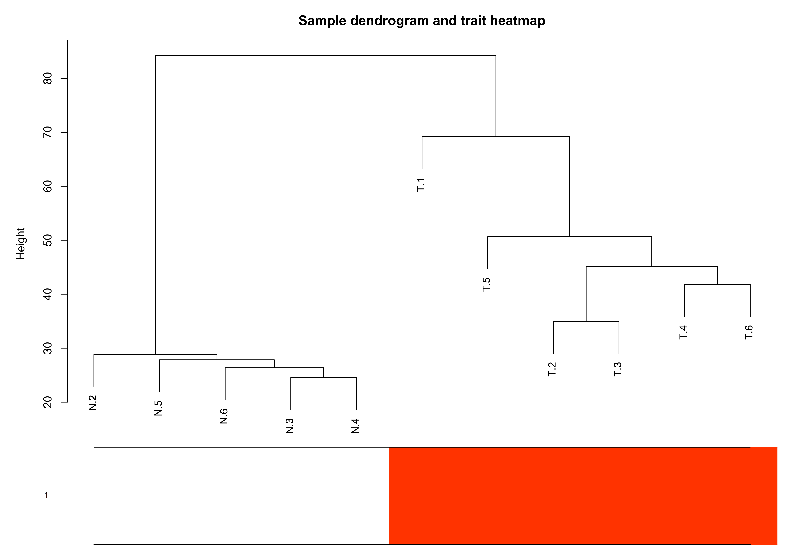


**A**

**C**

**B**

**Sample dendrogram and trait heatmap of GSE113513**

**Sample dendrogram and trait heatmap of GSE137327**

**Sample dendrogram and trait heatmap of GSE125961**

**FIGURE S2**


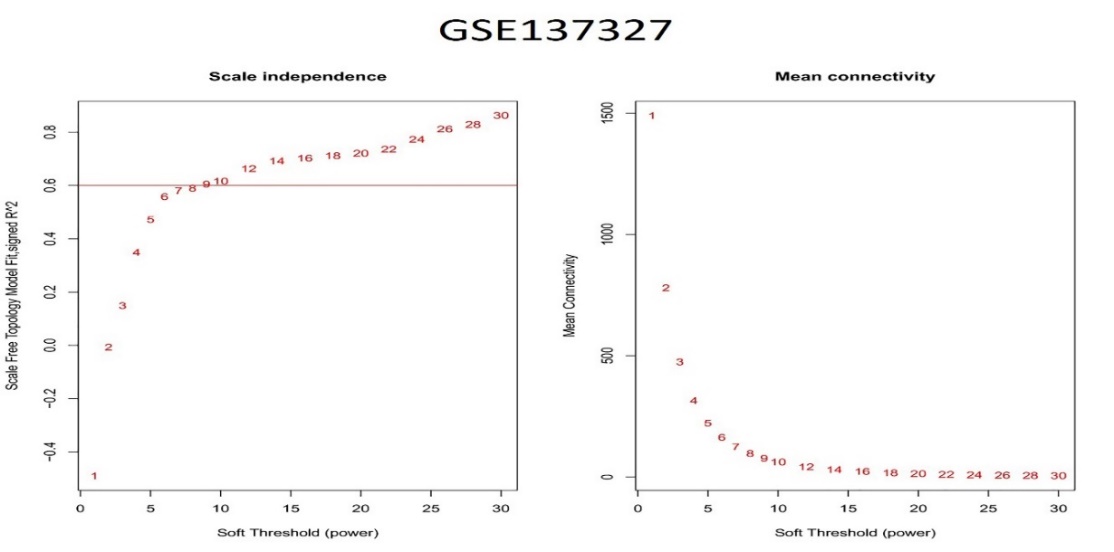

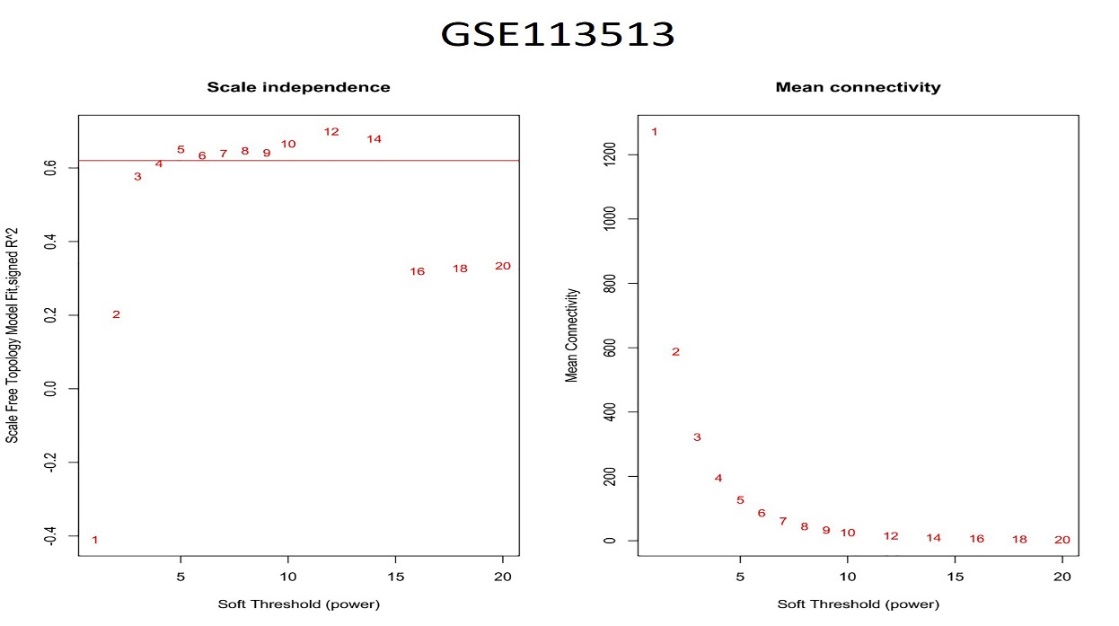

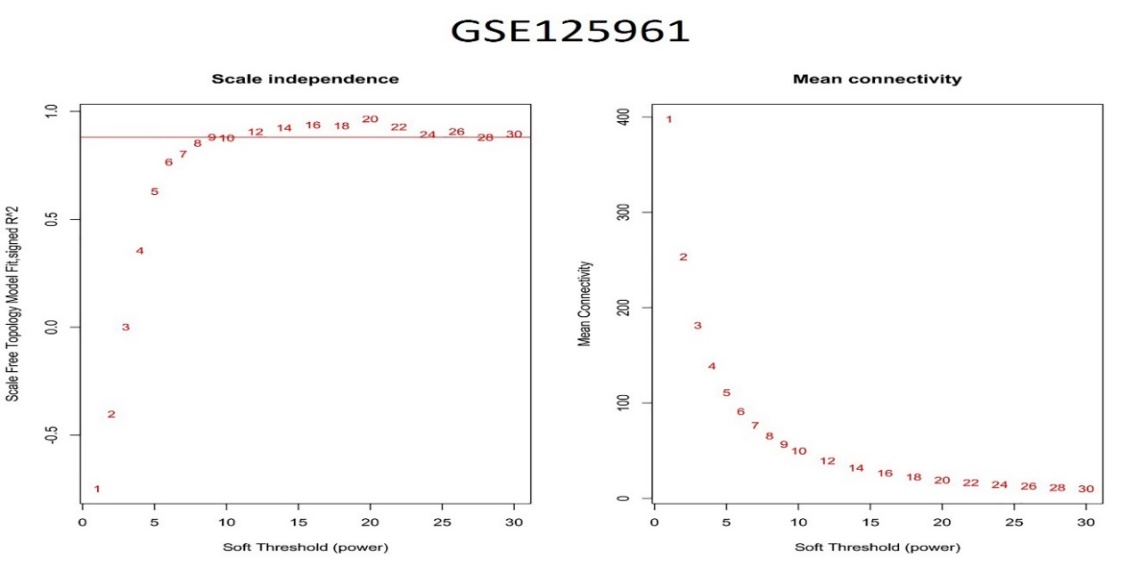


**A**

**B**

**C**

**GSE137327**

**GSE113513**

**GSE125961**

**Figure S4.** Selection of the soft-thresholding powers. The left panel shows the scale-free fit index (y-axis) as a function of the soft-thresholding power (x-axis). The right panel displays the mean connectivity (degree, y-axis) as a function of the soft-thresholding power (x-axis). **(A)** GSE137327; **(B)** GSE113513 and **(C)** GSE125961.

**FIGURE S3**


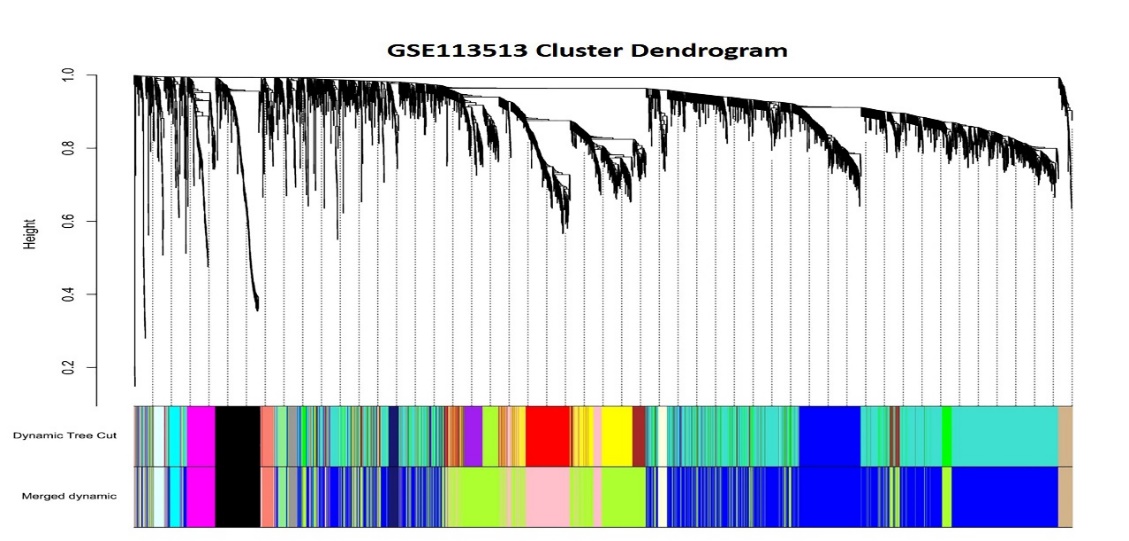

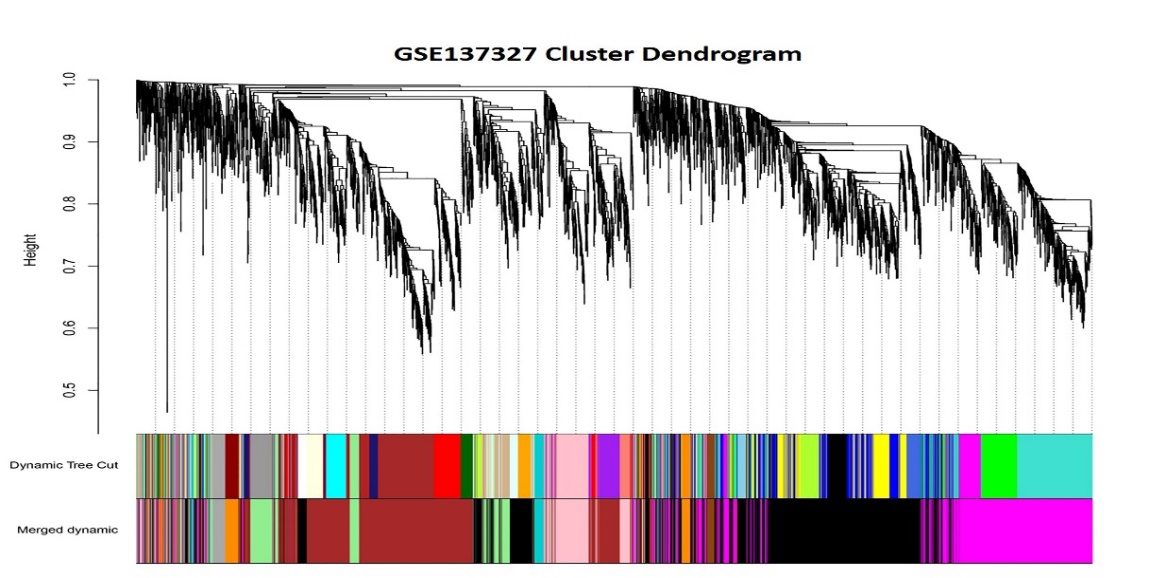

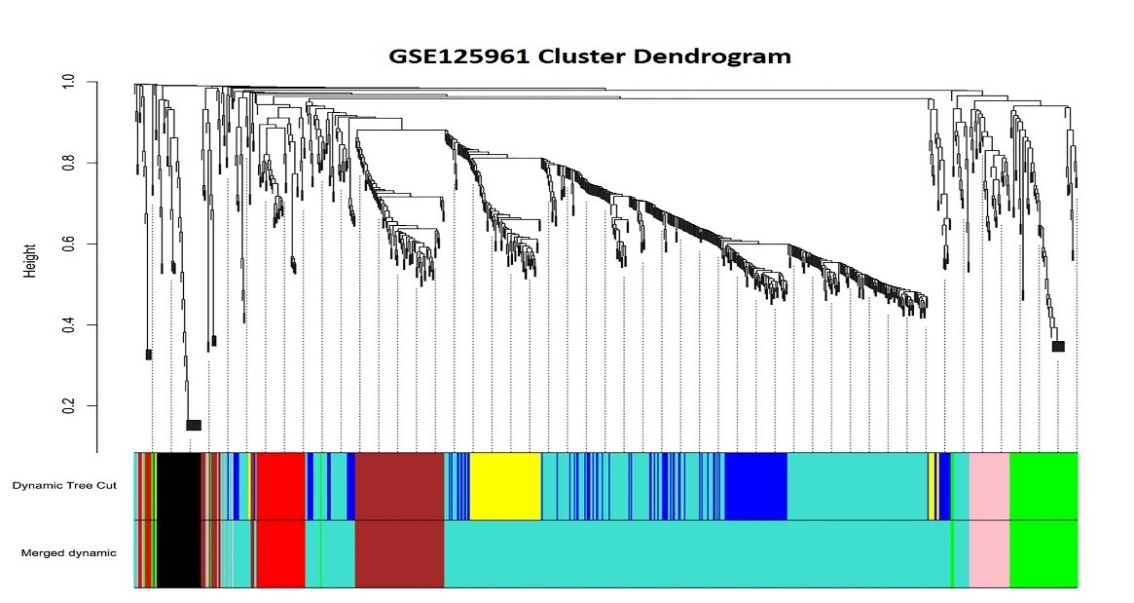


**A**

**B**

**C**

**Figure S3.** Cluster dendrogram and module assignment from WGCNA. The branches correspond to highly interconnected groups of genes. Colors in the horizontal bar represent the modules. **(A)** GSE137327; **(B)** GSE113513 and **(C)** GSE125961.

**FIGURE S4**

**Figure S4.** Module features of GS and MM (a) Modules significantly correlated with AA status (control vs. patient). Each point represents an individual gene within each module, which are plotted by GS on the y-axis and MM on the x-axis. **(A)** GSE137327 (blue module); **(B)** GSE113513 (black module); **(C)** GSE125961 (turquoise module) and **(D)** GSE125961 (brown module).


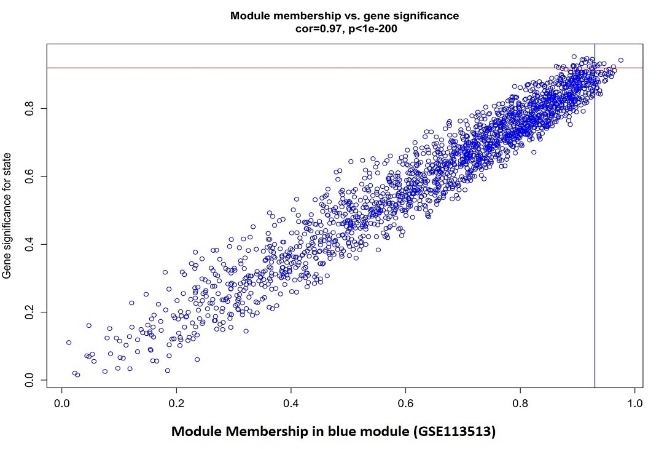

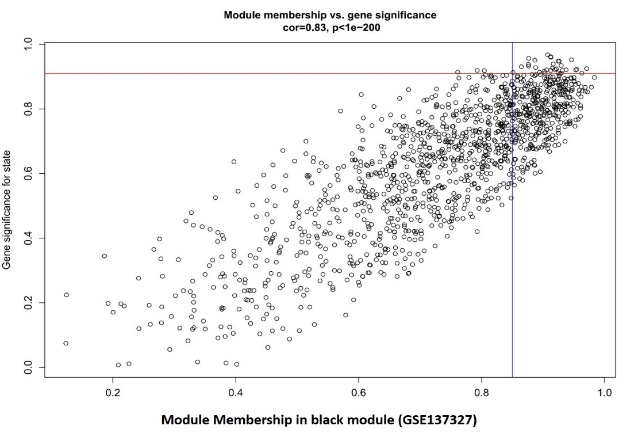

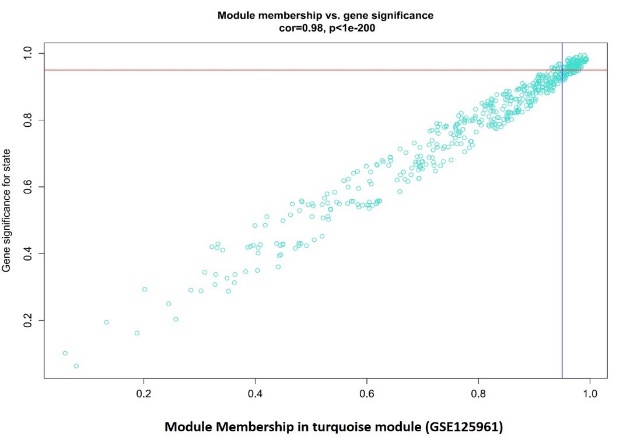


**A**

**B**

**C**


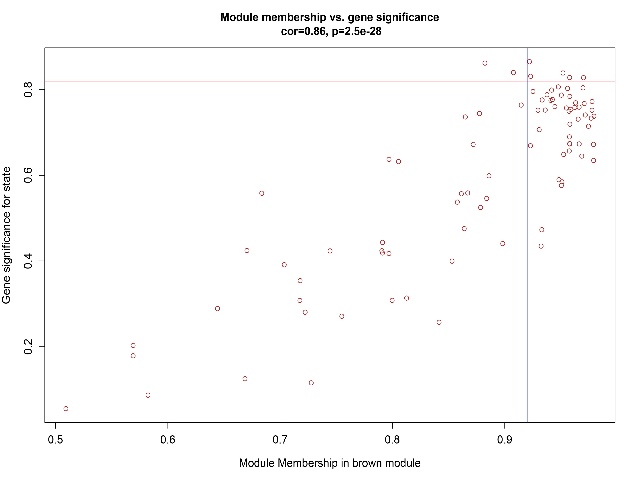


**D**

**Module Membership in brown module (GSE125961)**
