# Supplementary material for: Experimental validation of in silico analysis estimated the reverse effect of upregulated hsa‐miR‐106a‐5p and hsa‐miR‐223‐3p on SLC4A4 gene expression in Iranian patients with colorectal adenocarcinoma by RT‐qPCR
Source: Cancer Med. 2022 Dec 5;12(6):7005–18. doi: 10.1002/cam4.5499 (PMC10067115; doi:10.1002/cam4.5499)
Supplement: Supplementary file 2 — Table S1–S6 [file CAM4-12-7005-s002.docx]

**Supplementary Tables:**

**Table S1.** Datasets that used in this study.

**Table S2.** DEGs and DEMs analysis of selected datasets.

**Table S3.** Selected modules characteristics.

**Table S4.** The specific markers for different types of cells.

**Table S5.** Demographic information of tissue samples used for RT-qPCR analysis.

**Table S6.** The primer sequences used for RT-qPCR.

**Table S1**. Datasets that used in this study.

| **No.** | **Accession** | **Status** | **platform** | **Experiment type** | **Expression** | **No. samples** | **Ref.** |
| --- | --- | --- | --- | --- | --- | --- | --- |
| 1 | GSE137327 | 2019 | GPL23227 | high throughput sequencing | mRNA | 18 | [1] |
| 2 | GSE113513 | 2018 | GPL15207 | micro array | mRNA | 28 | [2] |
| 3 | GSE125961 | 2020 | GPL16791 | high throughput sequencing | miRNA | 12 | [3, 4] |
| 4 | GSE163974 | 2021 | GPL16791 | high throughput sequencing | mRNA  (Single-cell) | 2 | [5] |

**Table S2.** DEGs and DEMs analysis of selected datasets.

| Datasets | Type | #DEGs  (p-value < 0.001 and \|Log2FC\| ≥ 3) | | #DEMs  (p-value < 0.01 and \|Log2FC\| ≥ 1.5) | | Total |
| --- | --- | --- | --- | --- | --- | --- |
|  |  | **Up-regulated** | **Down-regulated** | **Up-regulated** | **Down-regulated** |  |
| GSE137327 | mRNA | - | 74 | - | - | 74 |
| GSE113513 | mRNA | 4 | 22 | - | - | 26 |
| GSE125961 | miRNA | - | - | 177 | 61 | 238 |

**Table S3.** Selected modules characteristics.

| Dataset | Module | Correlation | *p*-Value | #Genes |  | GS | MM | #Filtered Genes |
| --- | --- | --- | --- | --- | --- | --- | --- | --- |
| GSE137327 | Black | 0.87 | 6e-05 | 1275 |  | 0.91 | 0.85 | 31 |
| GSE113513 | Blue | 0.95 | 3e-14 | 1899 |  | 0.92 | 0.93 | 6 |
| GSE125961 | Turquoise | 0.99 | 3e-09 | 521 |  | 0.97 | 0.97 | 33 |
|  | Brown | 0.69 | 0.02 | 93 |  | 0.82 | 0.92 | 7 |

**Table S4.** The specific markers for different types of cells.

| Cell Type | Marker genes |
| --- | --- |
| Proximal Enterocytes | *ANPEP, FABP2, KRT20* |
| Distal Enterocytes | *RBP2, ANPEP, GPA33* |
| T-Cells | *CD8A, CD8B, CCL5, FOXP3* |
| B-Cells | *CD74, CD79B, MS4A1* |
| Paneth Cells | *DEFA5, DEFA6, REG3A, KRT20, SPIB* |
| NKT Cells | *EOMES, PRF1, NKG7* |
| Myeloid Cells | *JCHAIN, CLEC9A, CLEC10A* |

**Table S5.** Demographic information of tissue samples used for RT-qPCR analysis.

| Variables | | Number | % |
| --- | --- | --- | --- |
| Sex | Female | 8 | 42.0 |
|  | Male | 11 | 58.0 |
| Family history of cancer | Yes | 6 | 30 |
|  | No | 14 | 70 |
| Biopsy | Ascending colon | 1 | 5.0 |
|  | Cecum | 1 | 5.0 |
|  | Cecum &rectum | 1 | 5.0 |
|  | Descending | 2 | 10.0 |
|  | Rectum | 8 | 40.0 |
|  | Transverse colon | 4 | 20.0 |
|  | No report | 3 | 15.0 |
| Age | Mean ± SD | 61.45 ± 11.48 | |

**Table S6.** The primer sequences used for RT-qPCR.

| Gene | Forward primer (5' to 3') | Reverse primer (5' to 3') |
| --- | --- | --- |
| *SLC4A4* | CCGGCTTTGTTGGTCACTAT | CAAGTGATACCCTGCTCCTTTC |
| *GAPDH* | TGGACTCCACGACGTACTCAG | CGGGAAGCTTGTCATCAATGGAA |
| hsa-miR-223-3p | TGTCAGTTTAAATACCCCA | GAATCGAGCACCAGTTACGC |
| hsa-miR-106a-5p | ACCTCCTGTGTGCATGGATTA | GAATCGAGCACCAGTTACGC |
| U6 | GTGCTCGCTTCGGCAGCACATAT | GAATCGAGCACCAGTTACGC |

**References**

1. H, W. and Y. CS. Differential gene expression between human colon cancers and the adjacent nomral colon tissues. Gene Expression Omnibus (GEO). 2019. <https://www.ncbi.nlm.nih.gov/geo/query/acc.cgi?acc=GSE137327>.

2. J, P. and S. A. Gene expression profile of human colorectal carcinoma. Gene Expression Omnibus (GEO). 2018. <https://www.ncbi.nlm.nih.gov/geo/query/acc.cgi?acc=GSE113513>.

3. Y, D. and Z. F. Identification of miRNAs and Their targets in Colorectal Cancer (miRNA). Gene Expression Omnibus (GEO). 2020. <https://www.ncbi.nlm.nih.gov/geo/query/acc.cgi?acc=GSE125961>.

4. Zhou, F., D. Tang, Y. Xu, et al. Identification of microRNAs and their Endonucleolytic Cleavaged target mRNAs in colorectal cancer. BMC Cancer. 2020; 20:242. <https://doi.org/10.1186/s12885-020-06717-4>

5. H, W. and L. J. Colorectal cancer stem cell states uncovered by simultaneous single-cell analysis of transcriptome and telomeres. Gene Expression Omnibus (GEO). 2020. <https://www-ncbi-nlm-nih-gov.ezproxy.u-pec.fr/geo/query/acc.cgi?acc=GSE163974>.
